# Ciclesonide Inhaler Treatment for Mild-to-Moderate COVID-19: A Randomized, Open-Label, Phase 2 Trial

**DOI:** 10.3390/jcm10163545

**Published:** 2021-08-12

**Authors:** Joon-Young Song, Jin-Gu Yoon, Yu-Bin Seo, Jacob Lee, Joong-Sik Eom, Jin-Soo Lee, Won-Suk Choi, Eun-Young Lee, Young-Ah Choi, Hak-Jun Hyun, Hye Seong, Ji-Yun Noh, Hee-Jin Cheong, Woo-Joo Kim

**Affiliations:** 1 Division of Infectious Disease, Department of Internal Medicine, Korea University Guro Hospital, Korea University College of Medicine, Seoul 08308, Korea; infection@korea.ac.kr (J.-Y.S.); zephirisj9@gmail.com (J.-G.Y.); hak-neck@hanmail.net (H.-J.H.); msmjoonhoo@gmail.com (H.S.); jynoh@korea.ac.kr (J.-Y.N.); heejinmd@korea.ac.kr (H.-J.C.); 2 Division of Infectious Diseases, Department of Internal Medicine, Hallym University College of Medicine, Chuncheon 24252, Korea; yubinseo@gmail.com (Y.-B.S.); litjacob@chol.com (J.L.); 3 Division of Infectious Diseases, Department of Internal Medicine, Gil Medical Center, Gachon University College of Medicine, Incheon 21565, Korea; 386js@naver.com; 4 Division of Infectious Diseases, Department of Internal Medicine, Inha University College of Medicine, Incheon 22332, Korea; ljinsoo@inha.ac.kr; 5 Division of Infectious Diseases, Department of Internal Medicine, Korea University Ansan Hospital, Korea University College of Medicine, Ansan 15255, Korea; cmcws@korea.ac.kr; 6 Department of Internal Medicine, Seoul Metropolitan Government-Seoul National University Boramae Medical Center, Seoul 07061, Korea; Eunylee@kirams.re.kr; 7 Department of Internal Medicine, Seoul Metropolitan Seobuk Hospital, Seoul 03433, Korea; youngah12@seoul.go.kr

**Keywords:** COVID-19, SARS-CoV-2, ciclesonide, inhalation, antiviral agents

## Abstract

Although some intravenous drugs have been used to treat coronavirus disease 2019 (COVID-19), no effective antiviral agents are currently available in the outpatient setting. We aimed to evaluate the efficacy and adverse events of 14-day ciclesonide treatment vs. standard care for patients with mild-to-moderate COVID-19. A randomized, open-label, multicenter clinical trial of ciclesonide inhalers was conducted in patients with mild-to-moderate COVID-19. Patients were enrolled within 3 days of diagnosis or within 7 days from symptom onset and randomly assigned to receive either ciclesonide (320 µg inhalation twice per day for 14 days) or standard care. The primary endpoint was the severe acute respiratory syndrome coronavirus 2 (SARS-CoV-2) eradication rate on day 14 from study enrollment. Clinical status was assessed once daily, and serial nasopharyngeal viral load was evaluated by quantitative reverse transcription polymerase chain reaction. There were 35 and 26 patients in the ciclesonide and standard care groups, respectively. The SARS-CoV-2 eradication rate at day 14 was significantly higher in the ciclesonide group (*p* = 0.021). In multivariate analysis, SARS-CoV-2 negative conversion within 14 days was 12 times more likely in the ciclesonide group (95% confidence interval, 1.187–125.240). Additionally, the clinical failure rate (high-flow nasal oxygen therapy or mechanical ventilation) was significantly lower in the ciclesonide group (*p* = 0.034). In conclusion, ciclesonide inhalation shortened SARS-CoV-2 viral shedding duration, and it may inhibit the progression to acute respiratory failure in patients with mild-to-moderate COVID-19. Clinical Trial Registration NCT04330586.

## 1. Introduction

Coronavirus disease 2019 (COVID-19) presents several innate challenges, including insidious symptom onset, subclinical manifestations, and highly transmissible properties during the early stage of infection [1]. Thus, despite high-level public health interventions, COVID-19 has spread worldwide and has persisted since its first emergence in late December 2019. Cumulatively, more than 183 million people globally have been diagnosed with severe acute respiratory syndrome coronavirus 2 (SARS-CoV-2) infection, resulting in over 3.9 million deaths as of 5 July 2021 [2].

Antiviral drugs are used to improve clinical symptoms and ameliorate disease severity. Additionally, they have important clinical implications for suppressing disease transmission by reducing viral shedding duration. The development of antiviral drugs and repurposing of existing drugs are of great interest owing to limitations regarding compliance, inconvenience, and effectiveness of conventional public health measures such as wearing of mask, hand hygiene, and strengthened social distancing. Early effective antiviral therapy shortly after symptom onset may reduce viral shedding, thereby decreasing disease transmission. Hydroxychloroquine (HCQ), ritonavir-boosted lopinavir (lopinavir/r), and remdesivir have been investigated as drugs repurposed for the treatment of COVID-19 [3]. In the early stages of the COVID-19 pandemic, HCQ and lopinavir/r were expected to be effective repurposed drugs [4]. HCQ blocks endosomal acidification and inhibits viral uncoating, thereby inhibiting viral proliferation, while lopinavir/r is a protease inhibitor that inhibits enzymes that process 16 nonstructural proteins (NSPs) required for viral replication [4]. However, clinical trials of both these drugs (HCQ and lopinavir/r) have yielded disappointing results [5,6,7]. In a randomized clinical trial, intravenous administration of remdesivir, a polymerase inhibitor, significantly shortened the time to clinical recovery by 5 days but did not decrease the mortality rate [8]. Moreover, the currently available therapies for COVID-19 are injectables; thus, it is difficult to use them for patients with mild COVID-19 in outpatient clinics.

In comparison, ciclesonide (Alvesco^®^) is an inhaled steroid agent, which has been used to treat asthma. Although the mechanism is not yet clear, ciclesonide is presumed to exert antiviral effects by acting on the NSPs of SARS-CoV-2 [9]. Thus, ciclesonide is expected to have a dual effect (antiviral and anti-inflammatory effects) in the treatment of COVID-19. In case series reports from Japan, clinical symptoms and oxygen saturation improved when ciclesonide was administered to patients with COVID-19 pneumonia [10,11]. Based on favorable results from retrospective studies, randomized clinical trials have been conducted to evaluate the clinical efficacy of ciclesonide treatment for COVID-19 [12,13].

To evaluate the efficacy and adverse events of 14-day ciclesonide treatment vs. standard care for patients with mild-to-moderate COVID-19, we conducted a phase 2 randomized, open-label, multicenter study. 

## 2. Materials and Methods

### 2.1. Study Design

This randomized, open-label, multicenter clinical trial was conducted in six hospitals in South Korea from 8 May 2020 to 31 March 2021 (Clinical Trial Number—NCT04330586). Clade GH SARS-CoV-2 circulated dominantly (>90%) in South Korea during study periods. Patients (aged ≥19 years) with mild-to-moderate COVID-19, confirmed by quantitative reverse transcription polymerase chain reaction (qRT-PCR), were enrolled in the study within 3 days of diagnosis or within 7 days from symptom onset. Patients were eligible for the trial if they had a low National Early Warning Score (NEWS) ranging from 0 to 4. NEWS is a scoring system based on routine physiological parameters (respiratory rate, oxygen saturation, supplemental oxygen, body temperature, systolic blood pressure, heart rate, and level of consciousness), which can be obtained easily at the bedside. For each parameter, a score of zero is considered normal, and simple addition allows a total score from 0 to 20. A score of ≥5 represents the key threshold for urgent response, and patients with a score of ≥7 would be deemed to have a high-risk clinical condition requiring emergency response. Exclusion criteria included oxygen saturation <95% breathing room air, pregnancy or breastfeeding, renal impairment (estimated creatinine clearance <30 mL/min), hepatic dysfunction (alanine aminotransferase or aspartate aminotransferase levels more than five times the upper limit of normal), immunocompromising conditions, severe uncontrolled comorbidities, chronic airway diseases (asthma and chronic obstructive lung disease), and contraindications for use of ciclesonide inhaler. 

Eligible patients were randomly assigned in a 1:1:1 ratio to receive ciclesonide (320 µg inhalation twice per day for 14 days), ciclesonide-HCQ (320 µg inhalation twice per day for 14 days/400 mg daily for 10 days), or standard care. Expecting the synergistic or additive effect of ciclesonide and HCQ, the ciclesonide-HCQ combination was included in the comparison group. However, as data indicating that HCQ is not effective were published, the study design was altered to randomly assign patients to either ciclesonide or standard care groups. Thus, in the analyses, the ciclesonide-HCQ combination group was included in the ciclesonide group. Standard care comprised intravenous fluid, supplementary oxygen, and antibiotics, as necessary. The randomization was performed by computer-generated variable blocks ranging from 4 to 8 patients per each center, and the code numbers for eligible patients were assigned in ascending sequential order. Investigators of each hospital directly trained patients about the inhalation technique, providing educational materials to the patients. Even if symptoms improved, ciclesonide inhalation was maintained for 14 days.

The study was approved by the Institutional Review Board (IRB) of each participating hospital: Korea University Guro Hospital (2020GR0145), Hallym University Kangnam Sacred Heart Hospital (HKS2020-04-012), Gachon Gil Medical Center (GCIRB2020-152), Inha University Hospital (2020-04-023), Korea University Ansan Hospital (2020AS0085), Korea Cancer Center Hospital (KIRAMS 2020-04-002-002), and Seoul Metropolitan Seobuk Hospital (2020GR0145). In Seoul Metropolitan Seobuk Hospital, the study was conducted under the supervision of Korea University Guro Hospital IRB. The study was conducted in accordance with the Declaration of Helsinki and Good Clinical Practice, and all participants provided written informed consent prior to enrollment.

### 2.2. Clinical and Laboratory Monitoring

Enrolled patients were assessed once daily by the study investigators regarding symptoms and drug-related adverse events. Oxygen saturation was measured daily, and chest X-ray was taken weekly on day 1, 7 and 14. Serial nasopharyngeal samples were obtained on day 1 (before ciclesonide inhalation) and on days 4, 7, 10, and 14 for qRT-PCR until discharge. In addition, on day 3 (after inhalation of 320 µg ciclesonide four times) and day 4 (after inhalation of 320 µg ciclesonide six times) of study enrollment, saliva samples were collected from three study centers. The viral load (cyclic threshold (Ct) value) of SARS-CoV-2 from saliva was evaluated by qRT-PCR, and these were compared with the standard care control group. In each hospital, qRT-PCR tests for SARS-CoV-2 were conducted using test kits approved by the Korean Ministry of Food and Drug Safety, including Allplex™ 2019-nCoV Assay kit (Seegene, Seoul, Korea) and PowerCheck™ 2019-nCoV RT-PCR kit (KogeneBiotech, Seoul, Korea). Ct values of the RNA-dependent RNA polymerase (RdRp) gene were used for the assessment of viral load change. Clinical data were recorded in an electronic database and validated by the trial staff.

### 2.3. Outcome Measures

The primary endpoint was the SARS-CoV-2 eradication rate based on qRT-PCR on day 14 of study enrollment. SARS-CoV-2 eradication was defined as negative conversion of two consecutive negative results of qRT-PCR. Secondary endpoints were as follows: SARS-CoV-2 eradication rate based on qRT-PCR at days 7 and 10 from study enrollment; rate of clinical improvement (resolution of all systemic and respiratory symptoms) at days 7, 10, and 14 from study enrollment; rate of clinical failure within 28 days; safety/tolerability of ciclesonide. Clinical failure was defined as the case of clinical deterioration requiring high-flow nasal oxygen or mechanical ventilation, resulting in salvage treatment with dexamethasone and remdesivir.

### 2.4. Statistical Analysis

The original study sample size was estimated at 60, assuming that the virus eradication rate on day 14 after study enrollment would be 75% for the ciclesonide treatment group and 40% for the standard care group based on our previous clinical experience. This size sample would provide at least 80% power to detect a between-group difference at a two-sided significance level of α = 0.05. Considering the 10% dropout rate, 68 patients (34 per group) would be required.

Outcome analyses were conducted on an intention-to-treat basis, which included all patients who had undergone randomization. All ciclesonide-treated patients included in the analysis completed treatment by day 14 after enrollment. However, we excluded patients who withdrew consent, transferred to other hospitals within 7 days, and violated eligibility criteria. All statistical analyses were performed using SPSS (version 20.0; IBM Corp., Armonk, NY, USA). For categorical variables, univariate analysis was performed using either the chi-square test or Fisher’s exact test. Student’s *t*-test was used to compare continuous variables between the two groups and was expressed as median (interquartile range, IQR) or mean (standard deviation, SD). Statistical significance was set at *p* < 0.05. Multivariate analysis was performed to assess the independent contribution (odds ratio) of ciclesonide treatment for each clinical outcome using a logistic regression model; age, sex, underlying medical conditions, accompanying pneumonia, and Ct value at enrollment were adjusted.

## 3. Results

**Figure 1:**
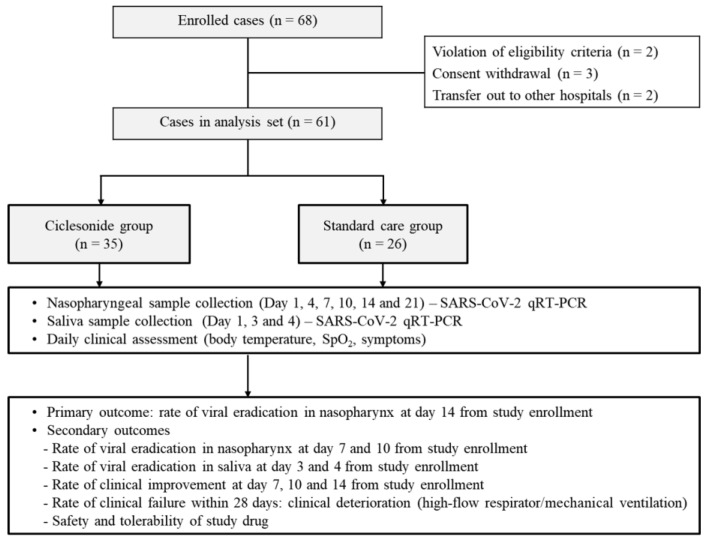
Study flowchart: randomization and treatment assignment.

Among 68 patients who underwent randomization, seven patients were excluded from the analyses because of issues with eligibility criteria (two patients), withdrawal of consent (three patients), or transfer to other hospitals within 3 days after study enrollment (two patients) (Figure 1). Among 61 patients in the analysis set, 35 patients were assigned to the ciclesonide group and 26 patients to the standard care group; eight patients in the ciclesonide group received oral HCQ treatment concomitantly for 10 days.

Patients’ median age was 53 (IQR, 35–61) years, and 47% were men (Table 1). The median interval from symptom onset to enrollment was 3 (IQR, 2–7) days, and the mean Ct value of the qRT-PCR for SARS-CoV-2 was 21.9 (standard deviation, 6.4) at study enrollment. At enrollment, no significant differences were found in demographics, underlying medical conditions, clinical manifestations, interval from symptom onset, Ct value, and NEWS between the two study groups (Table 1). Laboratory findings indicated that white blood cell counts were lower in the ciclesonide group than in the standard care group (3.262 vs. 4.493 cells/µL, *p* = 0.043), but all other lab tests were similar between the two groups.

Regarding the primary outcome, the SARS-CoV-2 eradication rate at day 14 was significantly higher in the ciclesonide group than in the standard care group (32.3% vs. 5.0%, *p* = 0.021) (Table 2). In the ciclesonide inhaler group, SARS-CoV-2 was negative-converted in 10 patients on the 14th day of treatment, and three of them received HCQ concurrently. Multivariate analysis revealed that SARS-CoV-2 was 12 times more likely to be eradicated at day 14 in the ciclesonide group than in the standard care group. Although not significant, SARS-CoV-2 eradication rates at days 7 and 10 were also higher in the ciclesonide group than in the standard care group. No significant between-group difference was observed in symptom-based clinical improvement rates at days 7, 10, and 14. However, the clinical failure rate was significantly lower in the ciclesonide group than in the standard care group (2.9% vs. 19.2%, *p* = 0.034). In the multivariate analysis, ciclesonide lowered the clinical failure rate by 97.4% (odds ratio 0.026; 95% confidence interval 0.001–0.845) compared with the standard care. No fatal cases were recorded in this study. Among non-pneumonic cases at study enrollment, pneumonia developed in 11.1% (3 of 27 cases) of ciclesonide group and 23.5% (4 of 17 cases) of standard care group, respectively (*p* = 0.273).

**Figure 2:**
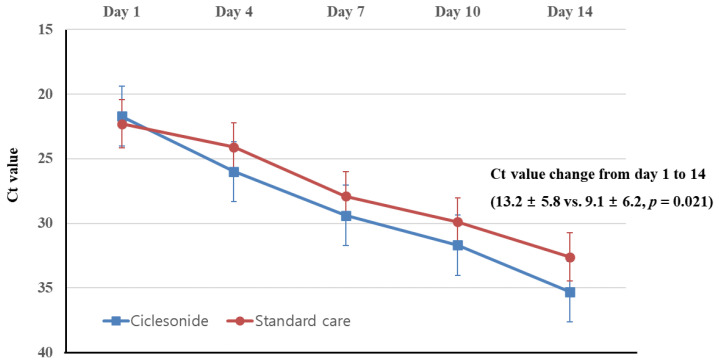
Comparison of serial cyclic threshold (Ct) values based on quantitative reverse transcription polymerase chain reaction targeting RdRp gene between ciclesonide and standard care groups. Four patients of ciclesonide group and six patients of standard care group were excluded in the analysis because of clinical failure or early discharge with clinical improvement, respectively.

When comparing the Ct values of nasopharyngeal specimens (Figure 2), no significant difference was found between the ciclesonide group and the standard care group at day 1 (21.7 ± 6.7 vs. 22.3 ± 6.1, *p* = 0.731), day 4 (26.0 ± 7.2 vs. 24.1 ± 5.5, *p* = 0.295), day 7 (29.4 ± 5.7 vs. 27.9 ± 5.5, *p* = 0.345), and day 10 (31.7 ± 5.1 vs. 29.9 ± 4.9, *p* = 0.226) from study enrollment, but the Ct value of the ciclesonide group on day 14 was marginally higher than that of the standard care group (35.3 ± 4.9 vs. 32.6 ± 4.2, *p* = 0.051). The change of the Ct value from day 1 to 14 was significantly larger in the ciclesonide group than in the standard care group (13.2 ± 5.8 vs. 9.1 ± 6.2, *p* = 0.021). If the qRT-PCR result was negative, the Ct value was assigned as 40.

For 22 patients, qRT-PCR was performed serially with saliva samples. When Ct values (mean ± SD) were compared between the ciclesonide group (*n* = 13) and standard care group (*n* = 9), no significant difference was observed at day 1 (28.5 ± 6.2 vs. 27.5 ± 6.8, *p* = 0.715), day 3 (31.4 ± 4.5 vs. 28.7 ± 5.5, *p* = 0.215), or day 4 (28.7 ± 4.1 vs. 29.8 vs. 6.1, *p* = 0.605).

Among the 35 patients who received ciclesonide, three complained of nausea, odynophagia, or headache after inhalation. These ciclesonide-related symptoms were tolerable, so treatment was continued for 14 days. The patient who had headaches received HCQ concomitantly. No serious adverse event was reported in any patients.

## 4. Discussion

This prospective, multicenter, randomized, open-label, phase 2 trial demonstrated that ciclesonide eradicated SARS-CoV-2 earlier and prevented the progression to severe COVID-19 among patients with mild-to-moderate COVID-19. Ciclesonide treatment increased the probability of SARS-CoV-2 negative conversion within 14 days by more than 12 times compared with standard care. Additionally, reduced risk of clinical failure (progression to hypoxia requiring respiratory management) by 97.4% was observed among patients who received ciclesonide compared with those who received standard care. However, in this study, we could not observe a significant shortening of symptom duration in the ciclesonide treatment group compared to the standard care group. The discrepancy may be due to the limitation of this study conducted in mild patients. Most mild symptoms other than fever are subjective, and self-limiting. Furthermore, because of individual variation, it is difficult to evaluate clinical improvement in mild patients. In order to obtain meaningful results, it would be necessary to evaluate patients with objective indicator (fever) in the acute stage within 48 h from symptom onset, as taken in the influenza study.

Inhaled ciclesonide can be safely delivered to lung tissues in high concentrations because it is essentially not absorbed into the bloodstream [14]. The antiviral mechanism of ciclesonide remains unclear. However, some studies have suggested that ciclesonide might suppress viral replication by inhibiting viral endoribonuclease (NSP15), p21 activated kinase-1, or viral RNA replication-transcription complex [9,12,15]. Ciclesonide is a prodrug that is converted to the active metabolite desisobutyryl-ciclesonide (des-CIC) by tissue esterases in the lung [15,16]. Although both ciclesonide and des-CIC are capable of interacting with NSP15, des-CIC has larger binding energy [9,15]. According to an in vitro study comparing diverse cell lines, the 90% effective concentration (EC90) of ciclesonide against SARS-CoV-2 was 10-fold lower (EC90 = 0.55 µM) in differentiated human bronchial tracheal epithelial cells than in VeroE6/TMPRSS2 or Calu-3 cells [9]. Considering that normal extravascular lung water may be <10 mL/kg, but increase with pulmonary edema, the EC90 for ciclesonide supports the administration of 640 µg/day (320 µg inhalation twice per day) in this study; 0.55 µM is equivalent to 1200 µg of ciclesonide dissolved in 4 L of exudate fluid [9,17].

During the early stage of infection, most cases of COVID-19 are mild, but 30–40% of patients experience pneumonia, and some rapidly worsen at approximately days 7–10. Thus, 14% require intensive care treatment and 5% become critical [18]. Therefore, even if the initial symptoms are mild, older and chronically ill patients should be closely monitored for possible worsening during treatment. Pathophysiologically, COVID-19 begins in the viral phase, passes through the immune (inflammation) phase, and then reaches the recovery phase. Some patients display acute exacerbation at 7–10 days of symptom onset, progressing to respiratory failure because of excessive inflammatory reactions. Given this, the corticosteroid dexamethasone appears to have a beneficial effect in patients with acute exacerbation of COVID-19 [19]. Ciclesonide is an inhaled corticosteroid used to treat bronchial asthma. Thus, in addition to its antiviral effect, the anti-inflammatory effects of ciclesonide may be useful in the treatment of lung injury, preventing progression to severe pneumonia and acute respiratory distress syndrome. Actually, favorable results have been reported in Japan when COVID-19 pneumonia cases were treated with ciclesonide inhalers [10,11]. Of note, in our study, the clinical failure rate due to acute respiratory failure was significantly lower in the ciclesonide treatment group than in the standard care group. Similar to our results, another inhaled glucocorticoid budesonide reduced clinical deterioration of mild COVID-19 by 91% in a randomized clinical trial [20].

Antiviral treatment for patients with mild COVID-19 requires consideration of two aspects: symptom relief and inhibition of viral transmission. A high SARS-CoV-2 viral load in saliva may contribute to efficient disease transmission in patients with mild COVID-19. Considering that ciclesonide is an inhalant, we expected a viral inhibitory effect in saliva during the early stage of infection, but contrary to expectations, the ciclesonide group did not display any difference in salivary SARS-CoV-2 suppression compared with the standard care group. Since ciclesonide is inhaled into the lower airways, the exposure time in the oral cavity is short, and the active metabolites generated in the lung tissue mainly exert antiviral effects [9,16]. Thus, ciclesonide inhalation may not sufficiently suppress salivary SARS-CoV-2. Given the antiviral effect of chlorhexidine, it may be effective to use a chlorhexidine gargle with ciclesonide to inhibit the excretion of SARS-CoV-2 from saliva during the early stage of infection [21]. Thus, it is necessary to evaluate whether this combined strategy is effective in blocking the transmission of SARS-CoV-2.

This study has some limitations. First, the trial was not blinded and was limited to a small sample size. Nevertheless, we recruited patients with COVID-19 during the early stage of infection within a mean of 3–4 days from symptom onset, and the baseline characteristics were comparable between the two study groups. Thus, the findings suggest the clinical usefulness of ciclesonide, but a larger, well-designed study is warranted to confirm our results. Second, viral culture tests were not conducted in this study, so the inhibitory effect of ciclesonide on viral viability could not be evaluated. Third, we evaluated viral shedding duration using two different Korean MFDS (Ministry of Food and Drug Safety)-approved qRT-PCR kits. Therefore, to minimize the effect of using two different kits, only one kit was used for each study participating institution, and block randomization was performed for each institution. Finally, data on the occurrence of secondary bacterial pneumonia and specific antibiotic treatment were not collected in this study.

In conclusion, our results indicate that ciclesonide shortened SARS-CoV-2 viral shedding duration. Ciclesonide may inhibit the progression to acute respiratory failure in patients with mild-to-moderate COVID-19. Ciclesonide inhalation could be a useful therapeutic option for mild-to-moderate COVID-19 in an outpatient setting.

## Figures and Tables

**Table 1:** Baseline demographic and clinical characteristics of study participants.

	Ciclesonide Group (*n* = 35)	Standard Care Group (*n* = 26)	*p*-Value
Age, mean days ± SD	44.9 ± 17.9	49.0 ± 16.8	0.362
Male sex, No. (%)	11 (31.4)	9 (34.6)	0.503
Underlying conditions (%)			
Diabetes	4 (11.4)	5 (19.2)	0.477
Hypertension	7 (20.0)	10 (38.5)	0.151
Cerebrovascular diseases	0 (0)	2 (7.7)	0.095
Clinical manifestations (%)			
Fever	17 (48.6)	12 (46.2)	0.852
Myalgia	16 (45.7)	12 (46.2)	0.973
Fatigue	11 (31.4)	7 (26.9)	0.781
Cough	20 (57.1)	10 (38.5)	0.198
Sputum	12 (34.3)	7 (26.9)	0.587
Sore throat	11 (31.4)	7 (26.9)	0.781
Rhinorrhea	7 (20.0)	4 (15.4)	0.745
Pneumonia (%)	8 (22.9)	9 (34.6)	0.391
Interval from symptom onset to enrollment, median days (IQR)	4 (2–7)	3 (1.8–5.5)	0.540
Ct value at enrollment, mean ± SD	21.7 ± 6.7	22.3 ± 6.1	0.731
NEWS at enrollment median (IQR)	0 (0)	0 (0–1)	0.519
Arterial oxygen saturation (%)	97.3 ± 1.5	97.5 ± 1.0	0.743
White cell count (cells/µL), mean ± SD	3262 ± 1934	4493 ± 2343	0.043
Hemoglobin (mg/dL)	13.9 ± 1.1	13.7 ± 1.4	0.617
Platelet count (cells/µL), mean ± SD	217 ± 63	206 ± 58	0.549
AST (IU/L), mean ± SD	26.0 ± 10.1	27.5 ± 18.4	0.677
ALT (IU/L), mean ± SD	23.7 ± 15.2	21.5 ± 18.3	0.610
BUN (mg/dL), mean ± SD	11.9 ± 3.6	13.6 ± 8.0	0.272
Serum creatinine (mg/dL), mean ± SD	0.8 ± 0.2	0.8 ± 0.2	0.964

ALT, alanine aminotransferase; AST, aspartate aminotransferase; BUN, blood urea nitrogen; Ct, cyclic threshold; IQR, interquartile range; NEWS, National Early Warning Score; SD, standard deviation.

**Table 2:** Comparison of clinical outcomes between ciclesonide and standard care groups.

	Ciclesonide Group (*n* = 35)	Standard Care Group (*n* = 26)	*p*-Value	Adjusted OR (95% CI) of Ciclesonide Treatment
Clinical failure rate, No. (%)	1 (2.9)	5 (19.2)	0.034	0.026 (0.001–0.845)
Clinical improvement rate at day 7, No. (%)	19 (54.3)	15 (57.7)	0.793	-
Clinical improvement rate at day 10, No. (%)	21 (60.0)	14 (53.8)	0.794	-
Clinical improvement rate at day 14, No. (%)	26 (74.3)	14 (53.8)	0.111	-
Virologic eradication rate at day 7, No. (%)	2/34 (5.9) ^a^	0/22 (0) ^b^	0.247	-
Virologic eradication rate at day 10, No. (%)	4/33 (12.1) ^a^	0/22 (0) ^b^	0.090	-
Virologic eradication rate at day 14, No. (%)	10/31 (32.3) ^a^	1/20 (5.0) ^b^	0.021	12.194 (1.187–125.240)
Duration of hospitalization, mean days ± SD	19.1 ± 7.7	19.5 ± 7.4	0.839	-

SD, standard deviation; OR, odds ratio; CI, confidence interval. ^a^ One patient was excluded at days 7, 10, and 14 because of clinical failure. In addition, one patient was excluded at day 10, and two more patients were excluded at day 14 because of early discharge with clinical improvement; respiratory specimens were not available. ^b^ Four patients were excluded at days 7 and 10, while five patients were excluded at day 14 because of clinical failure. In addition, one more patient was excluded at day 14 because of early discharge with clinical improvement; respiratory specimens were not available.

## Data Availability

The data are not publicly available because of ethical and regulatory restrictions on participant privacy. However, a pseudonymized individual study dataset will be made available on request directed to the corresponding author.
